# Spatial distribution and machine learning prediction of sexually transmitted infections and associated factors among sexually active men and women in Ethiopia, evidence from EDHS 2016

**DOI:** 10.1186/s12879-023-07987-6

**Published:** 2023-01-23

**Authors:** Abdul-Aziz Kebede Kassaw, Tesfahun Melese Yilma, Yakub Sebastian, Abraham Yeneneh Birhanu, Mequannent Sharew Melaku, Sebwedin Surur Jemal

**Affiliations:** 1 Department of Health Informatics, School of Public Health, College of Medicine and Health Sciences, Wollo University, Dessie, Ethiopia; 2 Department of Health Informatics, Institute of Public Health, College of Medicine and Health Sciences, University of Gondar, Gondar, Ethiopia; 3 Charles Darwin University, Casuarina, Australia; 4 Department of Statistics, Collage of Natural and Computational Science, Mizan Tepi University, MizanTepi, Ethiopia

**Keywords:** Sexually transmitted infections, Spatial distribution, Machine learning, Prediction, Ethiopia

## Abstract

**Introduction:**

Sexually transmitted infections (STIs) are the major public health problem globally, affecting millions of people every day. The burden is high in the Sub-Saharan region, including Ethiopia. Besides, there is little evidence on the distribution of STIs across Ethiopian regions. Hence, having a better understanding of the infections is of great importance to lessen their burden on society. Therefore, this article aimed to assess predictors of STIs using machine learning techniques and their geographic distribution across Ethiopian regions. Assessing the predictors of STIs and their spatial distribution could help policymakers to understand the problems better and design interventions accordingly.

**Methods:**

A community-based cross-sectional study was conducted from January 18, 2016, to June 27, 2016, using the 2016 Ethiopian Demography and Health Survey (EDHS) dataset. We applied spatial autocorrelation analysis using Global Moran’s I statistics to detect latent STI clusters. Spatial scan statics was done to identify local significant clusters based on the Bernoulli model using the SaTScan™ for spatial distribution and Supervised machine learning models such as C5.0 Decision tree, Random Forest, Support Vector Machine, Naïve Bayes, and Logistic regression were applied to the 2016 EDHS dataset for STI prediction and their performances were analyzed. Association rules were done using an unsupervised machine learning algorithm.

**Results:**

The spatial distribution of STI in Ethiopia was clustered across the country with a global Moran’s index = 0.06 and p value = 0.04. The Random Forest algorithm was best for STI prediction with 69.48% balanced accuracy and 68.50% area under the curve. The random forest model showed that region, wealth, age category, educational level, age at first sex, working status, marital status, media access, alcohol drinking, chat chewing, and sex of the respondent were the top 11 predictors of STI in Ethiopia.

**Conclusion:**

Applying random forest machine learning algorithm for STI prediction in Ethiopia is the proposed model to identify the predictors of STIs.

## Introduction

Sexually transmitted infections (STIs) are sexual organ-related infections caused by pathogens which are a variety of clinical sets of indicators transmitted through sexual activities and most of them are simply preventable and curable [1]. Worldwide there are above 30 well-known STIs caused by bacterial, viral, and parasitic pathogens that are recognized as transmitted through sexual contact [2]. STIs are one of the major public health problems worldwide that cause severe illness, long-term impediments, infertility, medical and psychological consequences as well as deaths [1]. Furthermore, STIs facilitate and assist the expansion of HIV/ADIS [3].

The majority of STIs are present without symptoms; Some STIs can increase the risk of HIV acquisition three-fold or more [4]. Newborn, women, and multi-sexual are highly risky for STI [5]. Many HIV prevention studies globally gather baseline data on STIs, this situation indicated a strong association between STIs and HIV [6].

According to the WHO reports an estimated 357 million new cases of curable STIs such as gonorrhea, chlamydia, trichomoniasis, and syphilis happened in 2012. Among these, 142 million are in southeast Asia, 64 million in America, 63 million in Africa, 39 million in the western pacific, 31 million in European, and 18 million in the eastern Mediterranean region. Of these, the majority occurred in developing countries [7]. Worldwide, millions of people acquire STI every day; of these, 376 million new cases of treatable Syphilis, Gonorrhea, Chlamydia, and Trichomonas occur every year and an estimate of 536 million people are living with incurable Herpes Simplex Virus Type 2 infection [8]. According to the 2018 CDC report, America had 2,457,118 Combined Cases. From these, Chlamydia 1,758,668 cases (540 per 100,000 people) Gonorrhea 583,405 cases (179 per 100,000 people) and Syphilis (all stages) 115,045 cases (35 per 100,000 people) [5].

In Ethiopia, the prevalence of abnormal genital discharge increased from 1.4 to 3% among women and 1 to 2% among men from 2005 to 2011 respectively. Likewise, genital sore increased from 0.8 to 1% among women and 0.4 to 0.7% among men [9, 10]. The 2013 STIs surveillance in Ethiopia indicated 50% vaginal discharge and 31% urethral discharge. It also showed that 16% of STI patients were co-infected with HIV, of these 8.1% and 21% were male and female respectively [10].

The 2016 national reproductive health strategy of Ethiopia identifies STIs prevention and control as one of the strategies to prevent and control HIV infection and the immediate and long-term complications of STIs [11]. A baseline data progress assessment is important to describe the STI burden, service delivery gaps, and opportunities at the national level [12]. However, in Ethiopia, limited studies have been conducted on STIs. Most of these have been done in a small-scale area and using small sample sizes. These studies also did not address the spatial distribution of infection in the geographic area of the study which is crucial for identifying the risky and non-risky areas that are important to improve and develop intervention and prevention mechanisms.

The accessibility and availability of huge amounts of data like EDHS would be able to provide us with useful knowledge when data mining techniques are applied to it. The rise in the machine learning approach could help to identify factors associated with STI in a comprehensive way [13]. Different machine learning techniques are useful for examining the data from diverse perspectives and summarizing it into valuable information.

Available research studies in Ethiopia focused on traditional statistical methods [14–16] to determine the relationship among variables, that are based on prior assumptions which could limit the potential to discover hidden knowledge. Machine learning models, on the other hand, are designed to make the most accurate predictions possible enabling systems to learn from data rather than making prior assumptions [17]. The need to develop a better STIs prediction model is essential for early screening. Since STIs are complex diseases, applying predictive modeling using a novel approach will provide a new insight into the disease thereby improving the care for the population. In this research, machine learning prediction methods will be applied to fill this gap. The methods have a mechanism that handles the imbalanced data which makes it biased in the traditional statistical regression model such as logistic regression and linear regression. Therefore, this research aimed to assess predictors of STIs using machine learning techniques and their geographic distribution across Ethiopian regions.

## Methods

A community-based cross-sectional study using EDHS 2016 dataset was conducted from January 18 to June 27, 2016. The study was conducted in Ethiopia, which is located in the Horn of Africa (30–150 N latitude and 330–480 E longitude) [18] and the headquarters of the African Union. Administratively, Ethiopia is divided into nine regions and two administrative cities. The country occupies an area of 1.1 million square kilometers and has an estimated 114,963,588 people at mid-year 2020. Of the total, 21.3% of the population live in urban and the remaining 79.7% lives in the rural part of the country [19]. In Ethiopia, there are 338 available and 218 under construction governmental Hospitals and 43 Private Hospitals, 4,063 available and 68 under-construction Health Centers, 3867 Private Clinics, 17,154 available, and 438 under-construction Health Posts were available [20].

The source populations were all sexually active men of (15–59 aged group) and women (15–49 aged group) living in Ethiopia. The study populations were all sexually active men and women in the selected Enumeration Areas (EAs) on EDHS 2016 dataset. All sexually active men and women who responded to previous self-reported STIs (yes/no) variables were included. Those respondents who did not have a sexual history before the survey were excluded. Ethiopia's demographic and health survey 2016 selected a total of 18,008 households for the sample, of which 17,067 were occupied. Of the occupied, 16,650 were successfully interviewed, yielding a response rate of 98%. The total household size was 16,650 and from this, 15,683 eligible women and 12,688 men were identified for individual interviews [21]. Among the interviewed men, 9038 had sex history before the survey and among the interviewed women, 11,916 had sex history. Then, merging these two extracted datasets gives a total of 20,954 with 155 missing values. After removing the missing variable, a total of 20,799 samples for STI prediction were included in this study.

The 2016 EDHS sample was stratified and selected in two stages. Each region was stratified into urban and rural areas, yielding 21 sampling strata. Samples of EAs were selected independently in each stratum in two stages. In the first stage, a total of 645 EAs (202 in urban areas and 443 in rural areas) were selected with probability proportional to each EA size. In the selection of the second phase, a fixed number of 28 households per cluster were selected with an equal probability of systematic selection from the newly created household listing. All women in the age group of 15–49 years and all men in the age groups between 15 and 59 who were either permanent residents of the selected households or visitors who stayed in the household at night before the survey were eligible to be interviewed [22]. All sexually active men and women who responded to previous self-reported STI (yes/no) question was included while those respondents who did not have a sexual history before the survey and zero coordinate values (zero longitudes and latitude) during spatial analysis were excluded.

The data analyses in this study had two stages. In the first stage, data relevance analysis like descriptive analysis and visualization was done using a statistical tool (R) and also converted the data to comma-delimited (CSV) format.

### Spatial autocorrelation and hot spot analysis

Spatial autocorrelation (Global Moran’s I) statistic measure was applied to determine whether STIs among sexually active men and women in Ethiopia are dispersed (Moran’s I values close to − 1), clustered (Moran’s I value close to + 1), or randomly distributed (Moran’s I value zero) in Ethiopia. Moran’s I value with (p-value < 0.05) suggests statistically significant spatial autocorrelation. Hot Spot Analysis of the z-scores and significant p-values tells the features with either hot spot or cold spot values for the clusters spatially. Hotspot Analysis is also used to identify the risked area in the geographical location on the study area. High-high clusters were used to investigate the local level cluster locations of STIs [23].

### Spatial scan statistics

Spatial scan statistics were used to identify significant local clusters. It also determines whether the observed patterns are due to chance or not. Spatial SaTScan statistics were applied based on the Bernoulli model using the Sat Scan™ software to analyze the purely spatial clusters of STI.

A Bernoulli-based model was used in which events at particular places were analyzed if the respondent had STI or not represented by a 0/1 value. A spatial SaTScan statistic uses a scan window (the population at risk) in the shape of a circle. Cluster size < 50 (maximum spatial default value) was used as an upper boundary. Then, clusters that are contained out of the maximum boundary with the window’s circular shape were ignored. ArcGIS 10.8 software was used to map the cluster and attribute of STI which was imported from SaTScan™ software [24].

### Spatial interpolation

The spatial interpolation technique was applied to predict unsampled data using the data feed by sampled data. The spatial interpolation analysis using the kriging technique was used to predict high-risk zones for STI in the study period [25].

The second stage was machine learning prediction which was performed using RStudio for data preprocessing like data cleaning and missing value handling [26]. Feature selection and variables importance rank [27, 28] was a technique for identifying a subset of features by removing irrelevant or redundant features. The importance of feature selection was to reduce the cost of learning by reducing the number of features. In this study, the Boruta algorithm was selected for feature selection [29]. Because the Boruta algorithm infers features’ relevance using the estimate of their importance from Random Forest and all feature is selected. It is also an all relevant feature selection algorithm.it is the ability to find both a strongly and weakly relevant variable features from the dataset [30].

After important features selected dividing the data into an explicit training dataset used to prepare the model [31] and an unseen test dataset used to evaluate the model's performance on unseen data was performed. Data balancing mechanism were used in this analysis because, As the name imbalanced data [32] indicated that when the data proportion in the outcome variable is disproportionate. If there is imbalanced data set in prediction, it will affect the result. So imbalanced data handling was applied in order to avoid biased prediction results.

The predictive modeling in machine learning is a modeling process where in predict the probability of an outcome using a set of predictor variables [33]. If the dependent variable is a binary response (yes/no), we could apply different classification machine learning algorithms [34]. So, machine learning prediction algorithms such as Random Forest, Naïve Bayes classifier, and Decision Tree (C5.0), Support Vector Machine with three different kernel and Logistic Regression was applied in this research. The prediction ability was practiced on both on a balanced and unbalanced dataset for each of the prediction algorithms.

Finally, the performances of the predictive models were evaluated via a number of standard evaluation metrics like Kappa statistics, ROC curve and accuracy with confusion matrix. Confusion matrix [35] is represented as

**Table:** 

N = Number of instances	Confirmed by observation	
	Yes	No
Predicted by test		
Yes	TP(Presence of disease)	FP(Type 1 error)
No	FN(Type 2 error)	TN(absence of disease)

*TP* true positive, *FP* false positive (type I error), *FN* false negative (type II error), *TN* true negative

True Positive Rate (TPR), False Positive Rate (FPR), Precision and Recall can be calculated as mentioned in Eqs. (1)–(6).

True Positive Rate (TPR) (sensitivity, Recall)

1 $${\text{TPR}} = {\text{TP}}/\left( {{\text{TP}} + {\text{FN}}} \right)$$

Precision

2 $${\text{Precision }}\left( {\text{positive predictive value}} \right) = {\text{TP}}/({\text{TP}} + {\text{FP}})$$

Negative Predictive Value/Rate

3 $${\text{Negative Predictive Value}}/{\text{Rate}} = {\text{ TN}}/\left( {{\text{TN}} + {\text{FN}}} \right)$$

Specificity (True Negative Rate)

4 $${\text{Specificity}} = {\text{TN}}/\left( {{\text{TN}}/{\text{FP}}} \right)$$

Receiver Operating Characteristics (ROC)

It is a trade-off curve drawn between True Positive Rate (TPR) and False Positive Rate (FPR).

5 $${\text{Accuracy (\% )}} = \left( {\left( {{\text{TP}} + {\text{TN}}} \right)\backslash \left( {{\text{TP}} + {\text{TN}} + {\text{FP}} + {\text{FN}}} \right)} \right) \times {1}00$$

6 $${\text{Balanced Accuracy}} = {1}/{2}\left( {{\text{sensitivity }} + {\text{ specificity}}} \right)$$

### Stages of machine learning algorithm general methodology (Fig. 1)

**Fig. 1:**
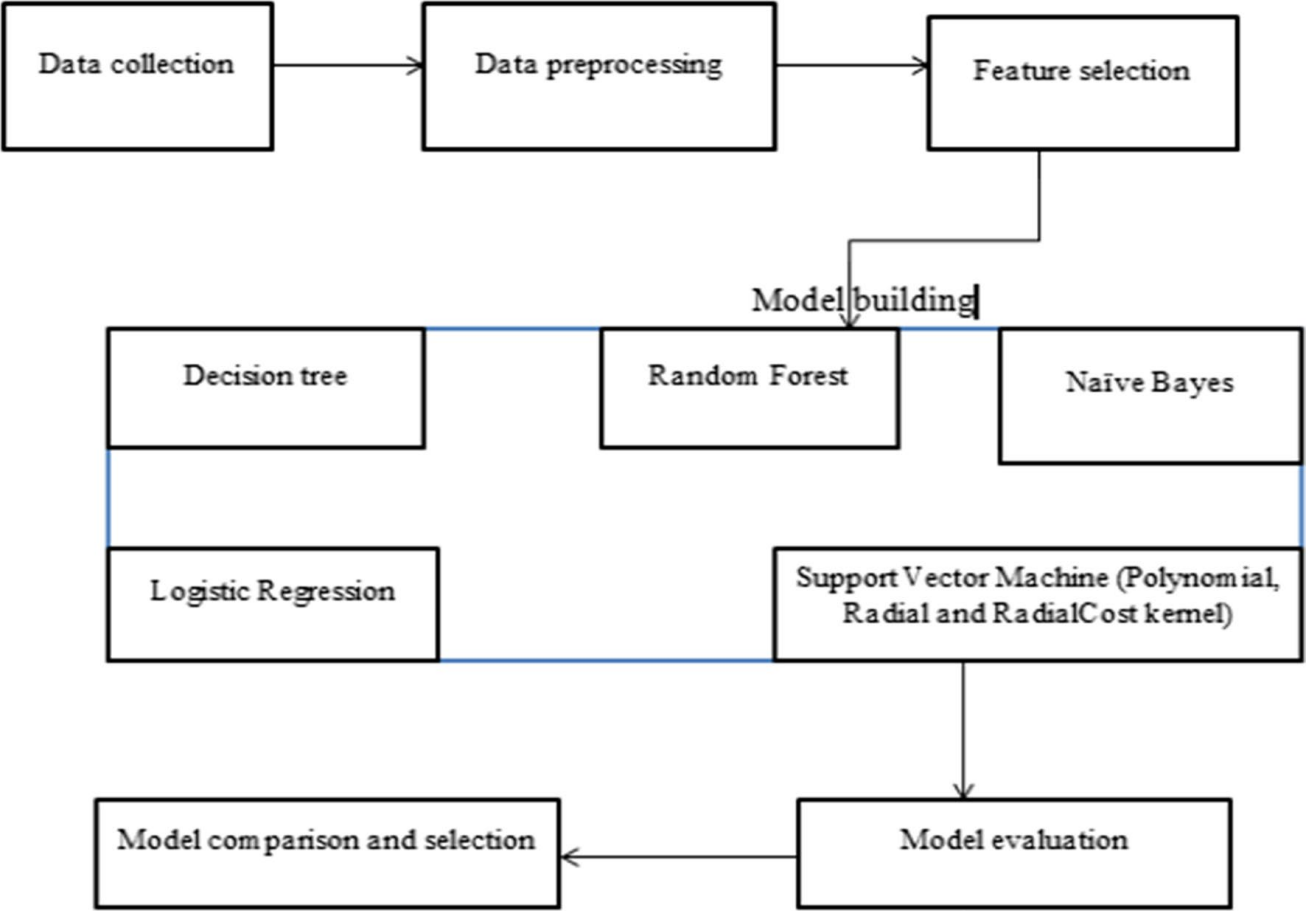
General information about methodology in machine learning for STI prediction

## Result

A total of 20,740 (weighted) sexually active men and women people were included in this study. The median age of the participants was 32 (IQR; 26, 40) years and most (39%) had under the age group of [25–34], A majority (57.9%) were females, 80.4% were from rural, most (81.6%) of respondent were married and more than half (60.1%) had their own work (Table 1).

**Table 1:** Frequency distribution of background characteristics of the study participants, EDHS 2016 (N = 20,740)

Variables	Category	Frequency (%)
Age of respondent	15–24	3960 (19.1)
	25–34	8094 (39.0)
	35–44	5695 (27.5)
	45–59	2991 (14.4)
Sex	Female	12,002 (57.9)
	Male	8738 (42.1)
Place of residence	Urban	4064 (19.6)
	Rural	16,676 (80.4)
Marital status	Not married	3814 (18.4)
	Married	16,926 (81.6)
Region	Tigray	1406 (6.8)
	Afar	1789 (0.9)
	Amhara	5219 (25.2)
	Somali	566 (2.7)
	Benishangul	223 (1.1)
	SNNPR	4084 (19.7)
	Gambela	66 (0.3)
	Harari	52 (0.2)
	Addis Ababa	1039 (5.0)
	Dire Dawa	118 (0.6)
Working status	No	8267 (39.9)
	Yes	12,473 (60.1)
Educational level	No education	10,371 (50.0)
	Primary	7018 (33.8)
	Secondary	1897 (9.1)
	Higher	1454 (7.0)
Wealth index	Poorest	3654 (17.6)
	Poorer	3992 (19.2)
	Middle	3990 (19.2)
	Richer	4085 (19.7)
	Richest	5019 (24.2)

### STI knowledge and information, sexual behavior and practice

The median age at 1st sex of the respondent was 18 (IQR; 15, 21) of these majority (65.7%) of the respondent had their first sexual intercourse aged <  = 19 years, nearly total (98.5%) had information about STI and Almost half (50.30%) of the study participant had media access (Table 2).

**Table 2:** Frequency distribution of STI knowledge and information, sexual behavior and practice of the study participants, EDHS 2016 (N = 20,740)

Variables	Category	Frequency (%)
Media_accesss	No	10,300 (49.7)
	Yes	10,440 (50.3)
Information about STIs	No	877 (4.2)
	Yes	19,863 (95.8)
Age at first sex	< = 19	13,620 (65.7)
	20 +	7120 (34.3)
Number of sexual partners excluding spouse	0 (No)	19,473 (93.9)
	1 (Single)	1093 (5.3)
	2 + (Multiple)	175 (0.8)
Chat chewing	No	16,234 (78.3)
	Yes	4506 (21.7)
Alcohol taking	No	11,911 (57.4)
	Yes	8829 (42.6)

### Spatial analysis

#### Spatial autocorrelation analysis of STI

The spatial autocorrelation is used to identify either the distribution of the disease was clustered, normal or dispersed. The spatial distribution of STI was not random in the study across Ethiopia. The spatial autocorrelation global Moran s I index of STIs in the 2016 EDHS survey was 0.06 and p-value: 0.04. The z-score of 2.0534 indicates that there is less than 5% chance that the clustered patterns are owing to random events.

#### Hotspot and cold spot (gets Ord Gi*) analysis of STI

**Fig. 2:**
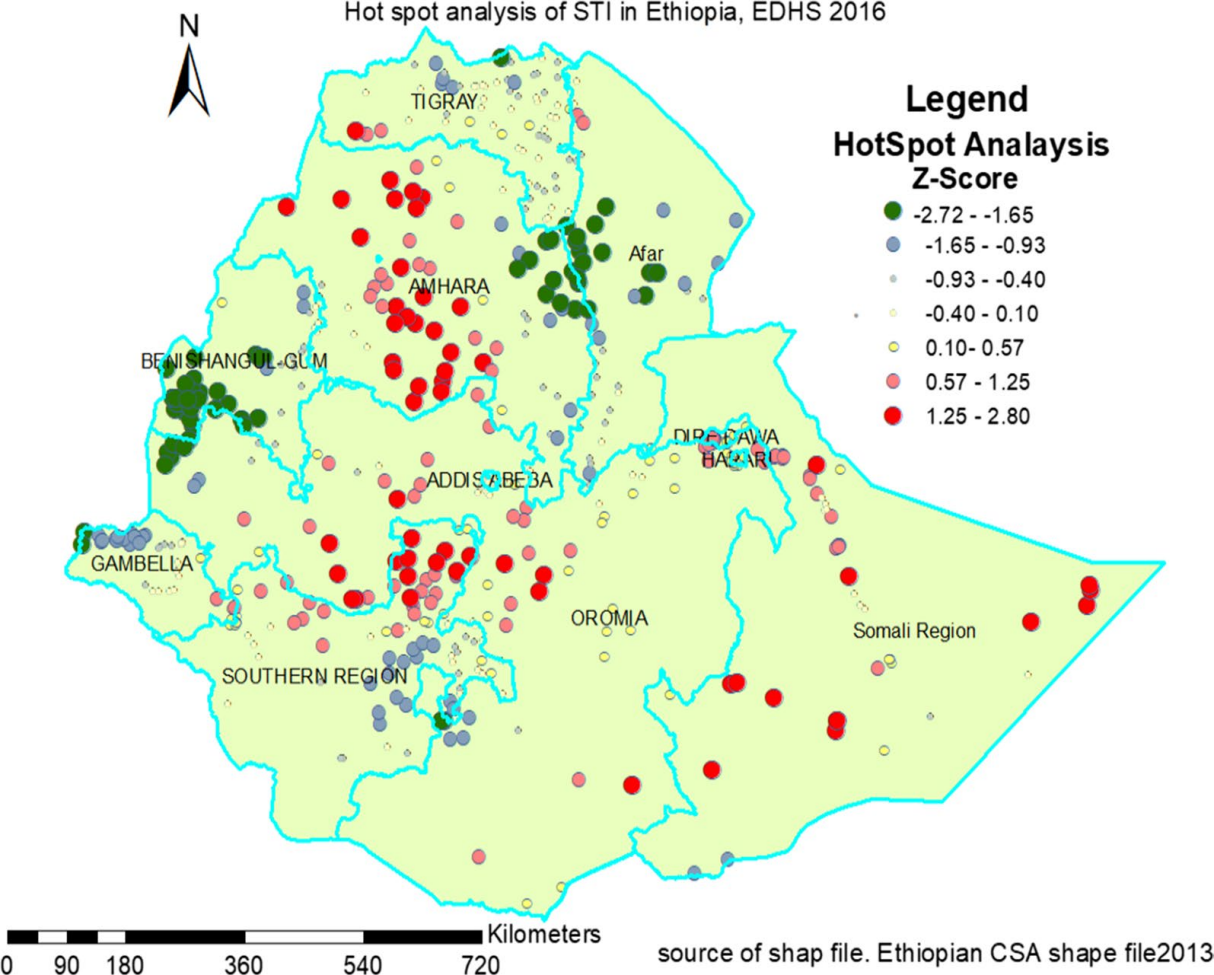
Hotspot analysis of STI among sexually active men and women in Ethiopia, EDHS 2016

The spatial autocorrelation result shows either the studied disease was dispersed, normal or clustered but it could not show in which study area that the case is found on areal study area. To identify this situation hot spot analysis was applied. The high proportion of STI in red color preceding the survey period in this study was detected in most parts of the Western Amhara and Southern Amhara regions; Central and Northwest Oromia regions; Eastern, Northern, and Southwest part of Somalia regions; Southwestern Tigray; and Harari Regional State. Whereas cold spots in green color was also detected in the Southwestern zone of Benishangul Region, Eastern zone of Amhara region, Western part of Afar regional state, and the Western zone in Gambela regional state of Ethiopia (Fig. 2).

#### Spatial scan statistics of STI

**Fig. 3:**
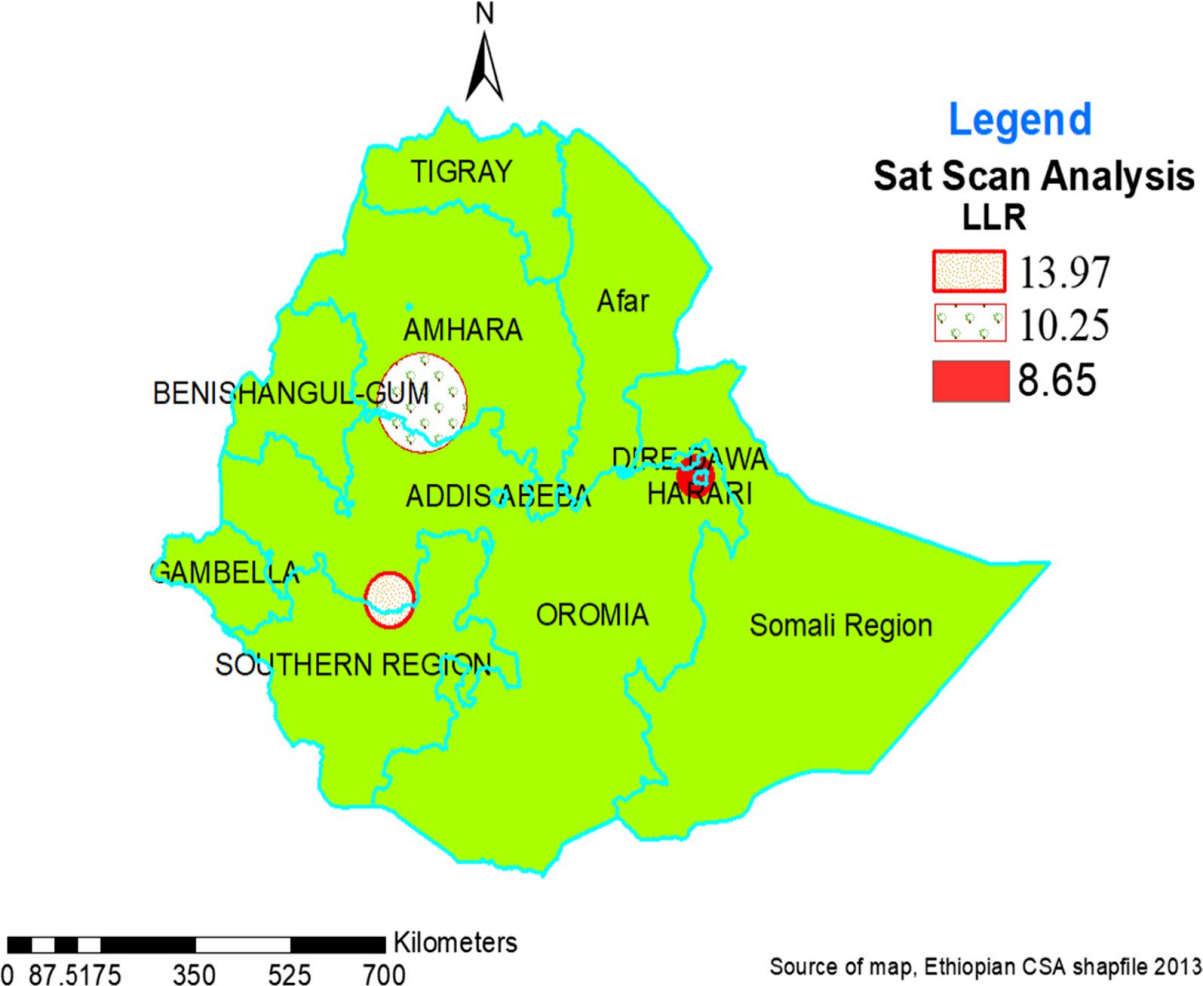
Sat Scan analysis of STI using the Kuldorff SaTScan approach among sexually active men and women in Ethiopia, EDHS 2016

In the SaTScan analysis, one primary (most likely) and two secondary significant clusters were detected (Fig. 3). The most likely primary clusters were located on the border of South West Oromia and the Northern part of the SNNR regional state. This spatial window was centered at 7.527086 N, 36.970948 E with a 45.13 km radius with a Relative Risk of 2.64 and log-likelihood Ratio (LLR = 13.97). It indicated that sexually active people inside the window were 2.64 times riskier for STI as compared with those found outside the window and Secondary cluster in the border of South West Amhara and North West Oromia regional state. This spatial window was centered at 10.477342 N, 37.499629 E with an 82.56 km radius (point data/single cluster), with a Relative Risk of 1.83 and log-likelihood Ratio (LLR = 10.25). It indicates that sexually active people inside the window were 1.83 times risker than outside the window. The remaining spatial window was another secondary cluster that was located in the Harari Regional State. This spatial window was centered at 9.370004 N, 42.102751 E with a 0 km radius (point data/single cluster), with a Relative Risk of 3.95 log-likelihood Ratio (LLR = 8.65). It indicated that sexually active people inside the window were 3.95 times riskier for STI as compared with those found outside the window (Table 3).

**Table 3:** Significant spatial scan statistics clusters of STIs among sexually active men, EDHS 2016

Cluster	Significant enumeration areas (clusters) detected	Coordinates/radius	Pop	Case	RR	LLR	P-value
Primary	447, 486, 227, 432	7.527 N, 36.971 E /45.13 km	412	39	2.64	13.97	< 0.001
2nd	474, 375, 531, 3, 218, 429, 24, 229, 482, 350, 403, 109, 120	10.477 N, 37.499E /82.56 km	1080	70	1.83	10.25	< 0.01
2^nd^	610	9.370 N, 42.103E /0 km	90	13	3.95	8.65	< 0.05

*2nd*  secondary, *Pop*  population, *RR*  relative risk, *LLR*  log likelihood ratio

### Spatial interpolation of STI in Ethiopia

**Fig. 4:**
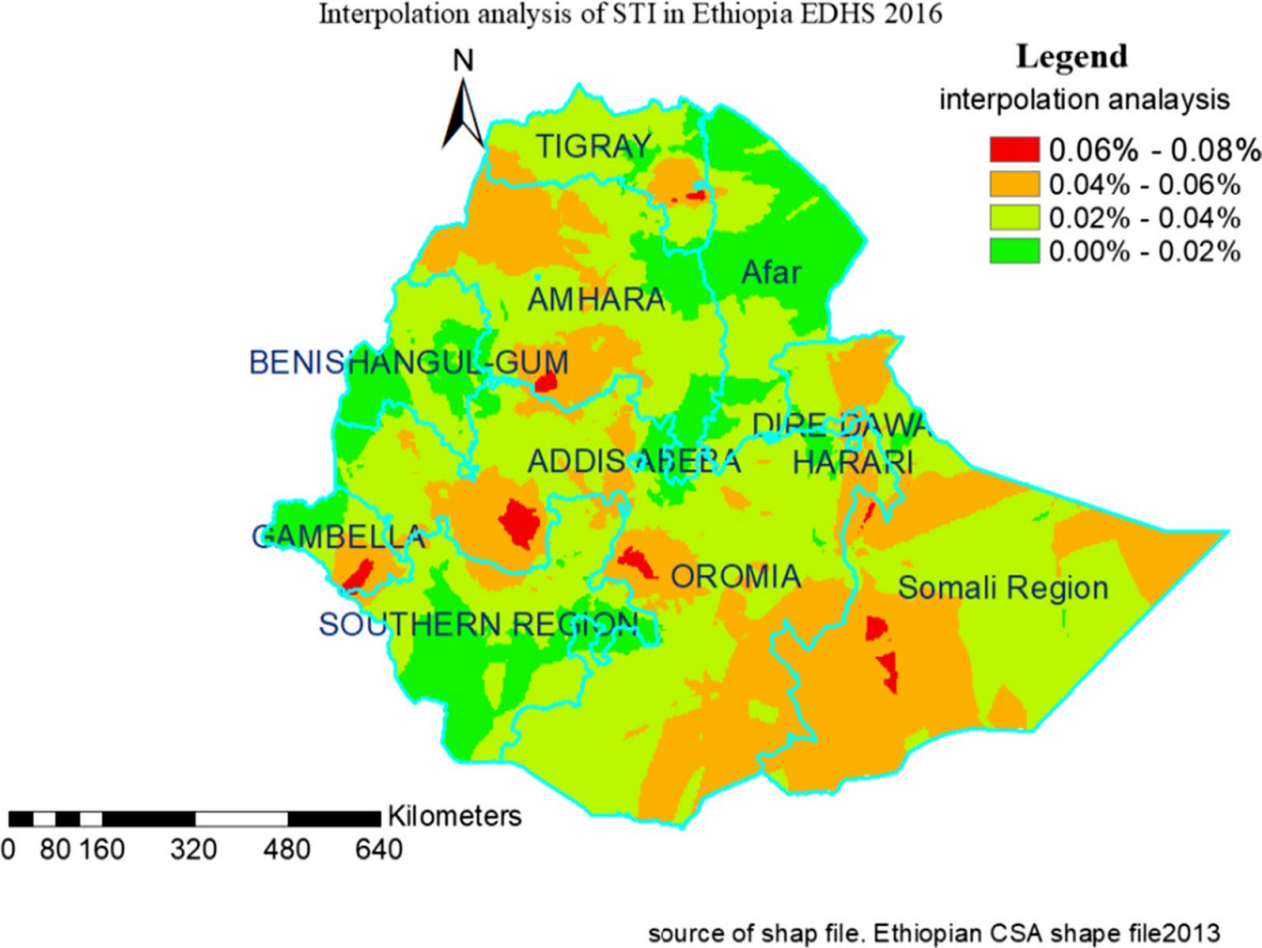
Kriging interpolation of STI among sexually active men and women in Ethiopia, EDHS 2016

The predicted STI prevalence over the area decreases from red to green-colored areas. The red color indicates the high-risk (high prevalence) of the STI predicted area and the green color indicates the predicted low-risk areas of STI prevalence. The kriging prediction map with red color told us that the Northwestern part of Gambela region, Southwest zone in Oromia region, Southwest and Northwest zone in Somalia region, part of Southwest zone in Amhara region, and South-Eastern zone of Tigray were predicted as risk areas for STI. While lowest STI prevalence rates were detected in the Eastern, Northern, and part of Northwestern zone in Afar regional state, part of Western and Eastern Benishangul-Gumuz, the Eastern part of Oromia from Addis Ababa in Oromia region, most of Southern zone in Amhara region, most of Southern, Southwestern and Eastern zone in SNNR of Ethiopia (Fig. 4).

### Feature selection method

**Fig. 5:**
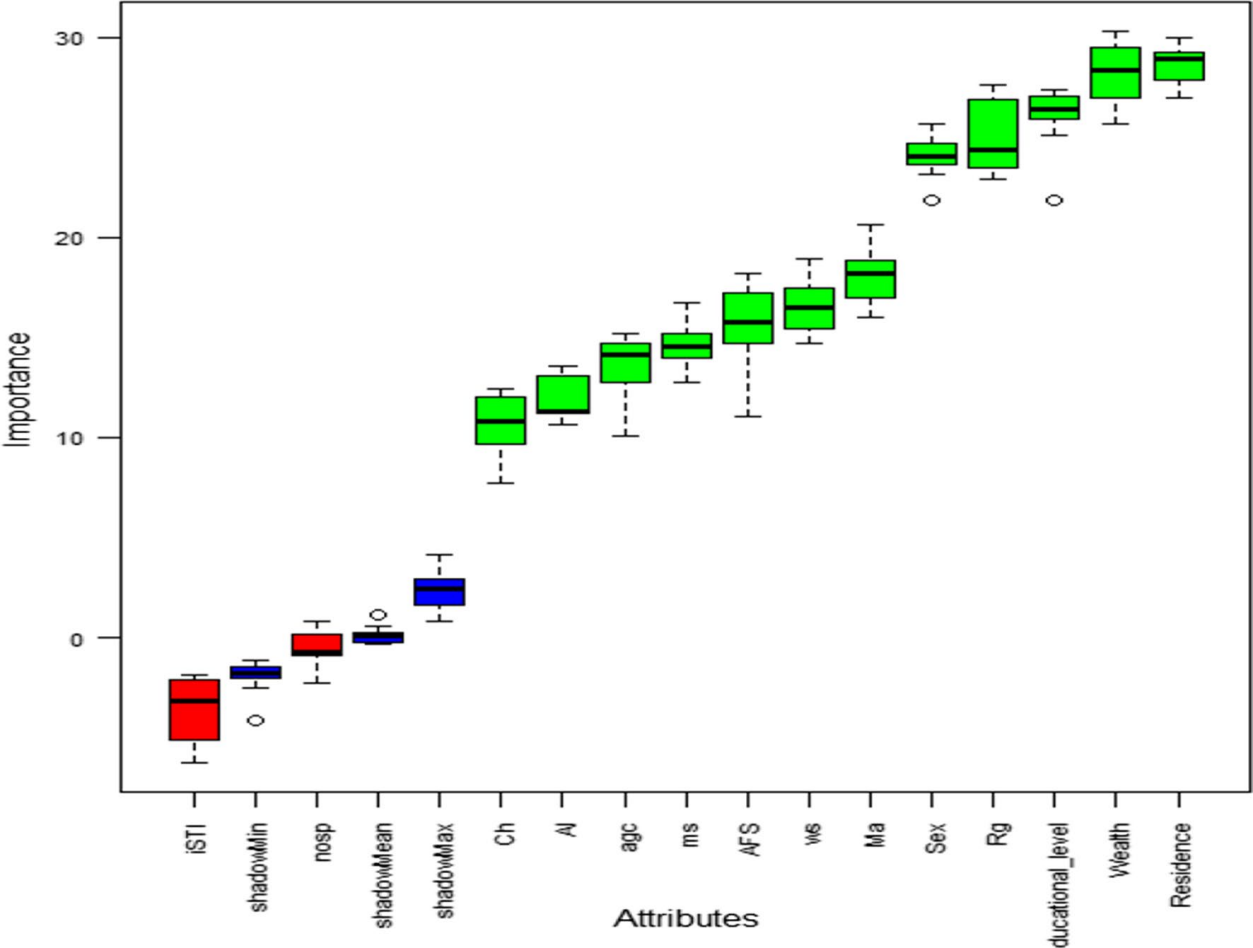
Important variable selection for STI prediction using Boruta algorithm, EDHS 2016. *iSTI*  information about STI, *nosp*  number of sexual partner, *Ch*  chat chewing, *Al*  alcohol drinking, *agc*  age group, *ms*  marital status, *AFS*  age at first sex, *ws*  working status, *Ma*  media access, *Rg*  region

The important feature from the data set was selected by using the Boruta algorithm which respects the outcome(dependent) variable "STI" by assigning the variables into two categories (confirmed and rejected) which can be viewed. Variables in the boxplot sorted by increasing importance and colored in green are those and greater than shadowmax which were classified as relevant and confirmed by the algorithm and Variables in red-colored are those and less than shadomax which are irrelevant and rejected by the algorithm (Fig. 5).

A balanced sampling method was applied to solve the imbalanced data problems and increase the performance of the machine learning algorithm. In the unbalanced dataset, 20,132 (96.8%) observations were under non case (No_STI) and 667 (3.2%) cases (STI).

Five balancing methods were applied to balance the unbalanced data for better prediction. These balancing methods are used to increase the performance of machine learning for disease prediction. Each balancing mechanism used its own technique for balancing the unbalanced data either by minimizing the majority class or maximizing the minority class (Table 4).

**Table 4:** Dependent variable distribution of STI before and after applying the balanced imbalanced data handling technique (methods)

Sampling methods	Class 1: (STI)	Class:2 (No_STI)	Total
Before balancing (unbalanced data)	667	20,132	20,799
	3.2%	96.8%	100%
Under sampling (balancing)	534	530	1064
	50.2%	49.8%	100%
Oversampling (balancing)	16,152	16,106	32,258
	50.1%	49.9%	100%
ROSE sampling (balancing)	8338	8302	16,640
	50.1%	49.9%	100%
Both under and over (balancing)	10,350	10,449	20,799
	49.8%	50.2%	100%
SMOTE sampling (balancing)	1068	1068	2136
	50%	50%	100%

**Fig. 6:**
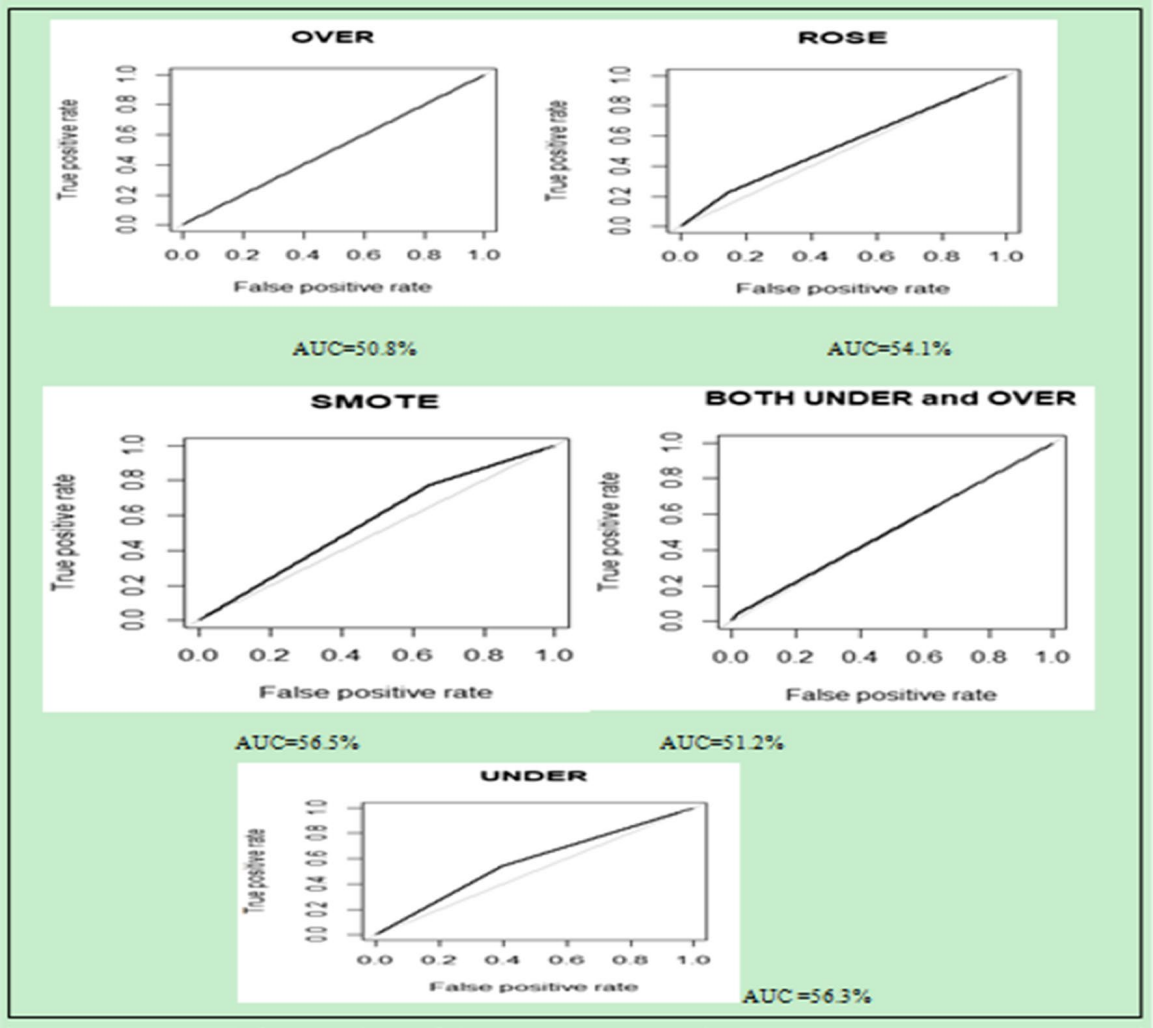
Performance measurement using AUC on all balanced sampling techniques

For comparison, the Random Forest classifier model was used and computed. However, performance measurement inaccuracy [35, 36] is not suitable for more unbalanced data because it measures within the specific point of action and it also depends on only the true positive and true negative observations this leads biased in the performance of the model because if the true negative observation is 99% the accuracy also greater than or equal to 99%. So, another performance measurement metric called ROC (receiver operating characteristics) curve or AUC (area under the curve) (Fig. 6) below, which is the rate of true positive and false positive predictive value was applied. The default value of AUC is 0.5 or 50%. If the value of AUC approaches’ 1, we call the performance of the machine learning prediction algorithm best. After five under and oversampling techniques were applied, the researcher got the following result to balance the unbalanced data. This technique is used to improve the performance of the model.

Therefore, depending on the above accuracy and AUC result of the sampling technique and the nature of the data originality. SMOTE sampling technique was the best of the rest for this research to balance the unbalanced data.

Supervised machine learning is a type of machine learning which trains a model with known inputs and output data to predict feature outputs [37]. It takes a known set of input data (the training set) and unknown responses to the output data. In this research, five supervised MLA classifier models were built to predict STI in Ethiopia such as the C5.0 decision tree, Random forest, Naïve Bayes algorithms, Logistic Regression, and Support Vector Machine. The models' prediction performances on the unseen dataset were measured and compared to the models' unbalanced and balanced train dataset.

All models were trained on a dataset of 80% and made a prediction on 20% of the unseen test dataset. The 80:20 data partition was applied on both the unbalanced and balanced data. RandomForest is the top one with a balanced accuracy of (69%), 65% sensitivity, 74% specificity, 72% positive predictive value, 68% negative predictive value, and 69% area under the curve (AUC) within (0.65–0.74) confidence interval in the balanced data set. The balanced accuracy of (50%), 0%sensitivity, 100% specificity, 97% negative predictive value, and 50% area under the curve (AUC) within (0.96, 0.97) confidence interval in the unbalanced data set (Table 5).

**Table 5:** Performance of supervised machine learning algorithm models for STI prediction in Ethiopia: evidence from EDHS 2016

Confusion matrix	Naïve Bayes		Random forest		Decision tree		SVM		LR	
	Observed		Observed		Observed		Observed		Observed	
	STI	No_STI	STI	No_STI	STI	No_STI	STI	No_STI	STI	No_STI
Predicted										
STI	120	90	138	55	122	82	123	80	110	106
No_STI	93	123	75	158	91	131	90	133	103	107
*Metrics*	*%*		*%*		*%*		*%*		*%*	
Accuracy	57		69		59		60		51	
95% CI	(52, 62)		(65, 74)		(55, 64)		(55, 65)		(46, 56)	
Sensitivity	56		65		57		58		52	
Specificity	58		74		62		62		50	
Positive predictive value	57		72		60		61		51	
Negative predictive value	57		68		59		60		51	
AUC	58		69		56		61		54	

**Fig. 7:**
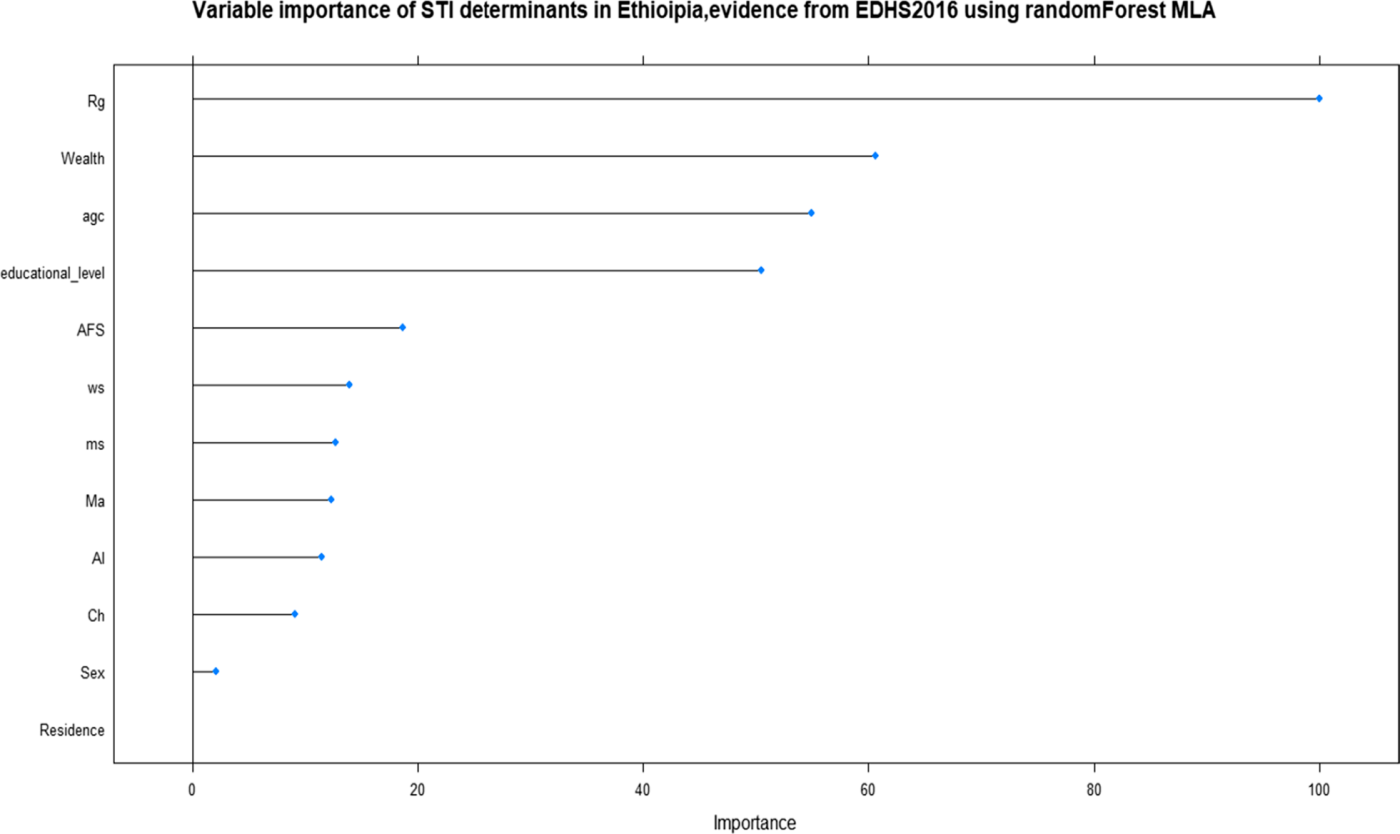
Variable importance measures of STI determinants in random forest algorithm, evidence from EDHS 2016. *Ch*  chat chewing, *Al*  alcohol drinking, *agc*  age group, *ms*  marital status, *AFS*  age at first sex, *ws*  working status, *Ma*  media access, *Rg*  region

Depending on the above (Fig. 7) overall result Random Forest classifier was the best classifier algorithm for STI prediction. For Random Forest classifier the selected features were ranked for STI prediction. The determinates were different magnitude for STI prediction The above figure (Fig. 7) showed as Region, wealth, age and educational status were the top most important (cause of STI) predictor variables. However, this ranking is not fully addressed the interrelationship between the predictors (selected independent variables) and outcome (STI) variable itself. As a result, we applied unsupervised machine learning. Unsupervised machine learning is the subset of machine learning which is used to find unknown patterns or essential structures in the input data (the hidden association between the predictors and the predicted one. In this machine learning, the users did not train or supervised the model. It discovers patterns and information that was previously undetected [37].

The association rules algorithm which is unsupervised prediction was applied in this Study. The rule-based prediction method generated 23 rules for classification or prediction. Among these, some of the important rules were extracted depending on the interesting rule performance measurement criteria. In this research, the researchers selected important rules based on the lift [65] which is an interesting quality measurement criterion of the association.

Lift/Interest is the ratio of the rule’s Confidence to the likelihood of occurrence of the resultant, which reflects a positive or negative correlation of if (antecedent) and then (consequence) of rules. It refers to the ratio of the occurrence probability of dependent/outcome variable (Y) under the condition of independent/explanatory variable (X) to that without considering condition X, which reflects the relationship between “X” and “Y”;

7 $$\mathrm{Lift }\left(\mathrm{X}\to \mathrm{Y}\right)=\frac{c(X\to Y)}{P(Y)}=\frac{P(X)}{P(X)P(Y)}$$

A range of lift values is [0, + ∞).

- As lift is equal to 1, it shows that X and Y appearing at the same time belong to independent random events and have no special significance; that means, X and Y are independent of each other with no mutual affection. We call these rules uncorrelated rule.
- If the lift value is less than 1, it shows that the occurrence of “X” reduces the occurrence of “Y,” and then we call them negative correlation rules (the association is protective rather than risk).
- If the lift value is larger than 1, it shows that the occurrence of “X” promotes the occurrence of “Y,” and then we call them positive correlation rules. Depending on this rule quality measure, the selected important rules were listed below (association rules).

- Rule 1: (lift 1.9) If a person who drank alcohol, lived in Afar, who was married, Then class STI = [0.94]
- Rule 2: (lift 1.6) If a person who chewed chat, lived either in Gambela, Harari, Addis Ababa or Dire Dawa, whose wealth index was either poorest or poorer, Then class STI = [0.79]
- Rule 3: (lift 1.5) If a person who did not chew chat, lived either in Harari, Addis Ababa or Dire Dawa, whose wealth index was richest, whose sex was female, who had media access, had married, whose age at 1st sex was less than 20, Then class STI = [0.76]
- Rule 4: (lift 1.4) If a person who lived Afar, Amhara, Oromia, or Somali, had work, was unmarried, Then class STI = [0.71]
- Rule 5: (lift 1.4) If a person who had drunk alcohol, lived in Afar or Amhara, had not to work), Then class STI = [0.69]
- Rule 6: (lift 1.4) If a person who lived in Ethiopia except in Tigray region, whose education level was either primary or secondary, whose education level was no education, primary or secondary, whose wealth index was richest, who had work, whose sex was female, who had media access, whose age at 1st sex was under 20, Then class STI = [0.68]
- Rule 7: (lift 1.2) If a person lived in either Oromia or Somali, Then class STI = [0.61]
- Rule 8: (lift 1.2) If a person who lived in Ethiopia except in Tigray, whose wealth status was richest, whose sex was female, who had media access, Then class STI = [0.61]
- Rule 9: (lift 1.1) If a person who chewed chat, who lived in Benishangul, SNNPR, Gambela, Harari, or Addis Ababa, Then class STI = [0.57]

## Discussion

In Ethiopia, the prevalence of STIs among sexually active men and women was found to be 3.2% with marked spatial heterogeneity. The spatial distribution result identified that the spatial variation of STI prevalence in Ethiopia was clustered which was confirmed by hotspot and SatScan analysis. Similarly, a study conducted in the US and China (clusters were located in the Yangtze River Delta region, the Southwestern border area of China) showed a regional variation of STI [38, 39]. This spatial variation could be explained due to the clustered effect of HIV ADIS prevalence in Ethiopia [40], and the health facility, urbanization, awareness, condom use, substance use, and unsafe sexual practice differences. If intervention measures were taken concerning the degree of variation, the prevalence and consequence of STI would be minimized.

After 14 variables were selected from the literature, the top 12 features were selected using the Boruta algorithm. The classifiers have been trained on a set of training samples with the imbalanced dataset. The result showed that the prediction balanced accuracy was low. Therefore, we understood that data balancing has a major influence on the prediction accuracy of the classifier because of this the researchers perform different resampling methods for data balancing.

Finally, SMOTE sampling technique was the selected one based on its AUC value. The most noticeable change balanced dataset is the significant increase in the average or balanced accuracy of the classifiers which is the average result of sensitivity and specificity and AUC. In the STI prediction analysis model, we understand that the entire seven models have similar prediction accuracy in data imbalance. However, when we look at the balanced accuracy and AUC in the balanced data set, random forest model was greater than the other and it is a good classifier model.

Therefore, the random forest was the best predictor model in this study with a performance evaluation of 97% accuracy, 50% average accuracy, and 50% AUC in imbalanced train data and 69% accuracy, 69% average accuracy, and 69% AUC on the balanced (SMOTE) data. This overall finding is in line with findings from the USA [41, 42]. However, there is a slight difference between these findings and finding from the USA. This might be due to the socio-economic, culture, lifestyle, and study area differences.

From the model performance evaluation result in this paper, we understand that data imbalance was an impact on the performance of prediction on the unseen new dataset. The random forest model shows a higher predictive power compared to the other machine learning algorithm models. In this regard, the random forest machine learning algorithm model showed that region, wealth, age category, educational level, age at first sex, working status, marital status, media access, alcohol drinking, chewing chat, and sex of the respondent were the top 11 predictors of STI in Ethiopia.

In this study, ‘region’ was found to be a factor for STI. This finding is supported by a research study conducted in Ethiopia [14]. This might be due to differences in the socio-demographic characteristics of the participants in each region. In this analysis, 57.6% of respondents from the Harari region were Chat abused which was much higher than in the other Ethiopian regions. The second reason might be due to alcohol consumption differences in the region. In the current analysis, 80.2% from the Amhara region reported that there were alcohol drinkers which was much higher than 0.6% in the Somali and 7.3% in the Afar region. The other reason might be the regional government and concerned body commitment differences in the country.

In this study, marital status was found to be a factor for STIs. This finding is supported by a research study in Swaziland [43]. This might be due to the behavioral and awareness difference between married and unmarried persons in relation to multiple sexual partners and substance use. Media access (exposure) was the other factor found to be associated with STI. This factor was also found to be a factor for STIs in research studies in Ethiopia [14], Ghana [44], and sub-Sahara Africa [45]. This might be due to the differences in the respondent’s media access and media program choices. The other reason could be due to the media broadcasting information about STIs prevention [14].

The other identified factor which is associated with STIs was the working status of the respondent. This finding is supported by studies conducted in sub-Sahara Africa and Swaziland [43, 45]. This could be due to different working area exposures [46]. The other reason could be the nature of the working area where worker in sex workers, bars, and nightclubs which is more vulnerable to unprotected causal sex [47].

Respondents who chewed chat were more likely affected by STI this was in lined with a study in Bahr Dar, Ethiopia [48]. This is due to the strong relationship between Chat Chewing and multiple sexual partner and It also leads to risky sexual practice [48]. Avoiding and controlling the availability of chat chewing is a mechanism to pr event and control the spread of STIs.

Alcohol consumption was found to be the other predictor identified in this study. This result was in line with research studies conducted in Harari and Gondar, Ethiopia [49, 50]. The possible explanation for the association of alcohol consumption with STIs could be due to the strong relationship between alcohol consumption and risky sexual practices [51]. That is, alcohol consumption before sex leads to inconsistent use of condoms and unsafe causal sexual practices [52]. Controlling and avoiding alcohol consumption is valuable to control and prevent STIs as well as HIV AIDS. The age of the respondents was also found to be a predictor of STIs. This result is supported by a study conducted in Swaziland [43]. This may be due to susceptibility difference of risky sexual behavior, immune deficiency development of STIs, and the number of sexual partners in the age group of the respondents could make age is factor for STIs [53].

Sex, wealth index, and age at 1st sex were also found to be the predictors that affect STIs. These results are also supported by different studies conducted in Sub-Saharan Africa, Swaziland, and France [54–56]. This might be due to the difference in awareness about STI prevention, unsafe sexual practices, and sexual violence. The other reason might be the vulnerability of women respondents to different socio-economic, biological, and cultural influences [5]. However, in this research, the results in the association rules showed us factors that associated with STI were different in different areas and magnitude depending on the situation. These are clearly expressed by the association rules of unsupervised prediction results.

(Rule 1) indicated that**,** If a person drinks alcohol, lives in Afar, and is married, then there will be a 94% chance of being affected by STI. This result pointed out that most of the respondents in the Afar region who drank alcohol were more vulnerable to STIs, especially those who had married. This might be due to the ability of alcohol pushes to do something emotionally; it may lead to causal unprotected sexual practice [57, 58]. The main cause of STIs in the Afar region was alcohol consumption. So, preventing the alcohol drinking practice before sex may be a solution for STIs prevention.

(Rule 2) indicated that if a person chews chat, lives either in Gambela, Harari, Addis Ababa, or Dire Dawa, and has a wealth index of either poorest or poorer, then there will be a 79% chance of being affected by STIs. This result revealed that Chat addictiveness and poorer and poorest economic levels of the respondents in these regions were the main factors for STIs. This might be due to the availability of chat in those regions (56.7% in Harari and 54.2% in Dire Dawa) which were much higher than the comparative (12.2% in Amhara and 2.9% in Tigray). This (Rule 2) result could be due to the relationship between chat chewing, alcohol consumption, and multiple sexual partners [51].

(Rule 3) indicated that if a person lives either in Harari, Dire Dawa, or Addis Ababa, has a wealth index of richest, has media access, is married, and whose age at first sex is under 20, then there will be a probability of 76% being affected by STIs. This result pointed out that in these regions, the main causes of STIs were being rich, availability of media, being married, and having sex in the age group of under 20 years. This could be due to the wrong choice of media programs like pornography and sex-related films [48, 51]. This could lead to early initiation of sex and unprotected sexual practice [59]. The other reason might be due to female adolescents wanting to complete most of the needs of peers concerning materials needs (fashion shoes, clothes, and electronics); this pushes them to early and unwanted sexual practice with older rich men to fulfill their needs [60–62]. Creating awareness about STIs, media program choice, and preventing early initiation of sex would be the solution to minimize and control the spread of STIs in these regions.

(Rule 6) showed that If a person lives in Ethiopia except Tigray**,** whose education level is either primary or secondary, whose wealth index is richest, who had a job, whose sex was female, who had media access, and whose age at 1st sex was under 20, then there will be a probability of 68% of being affected by STIs. This result revealed that being primary or secondary educational level, being richest, having a job, being female, having media access, and starting sex before the age group of under 20 years were the factors that affect STIs. This could be due to poor awareness about STIs [51], working area exposure due to working in high-risk areas such as sex workers, bars, guest houses, food facilitators, and similar facilities, sexual violence, and early initiation of unsafe sexual practices are at high risk of STIs [63]. The other reason may be due to the easy access to morally unaccepted media programs which makes to initiate unsafe and early initiation of sex by those who are the richest people. This is because if a person is rich, she/he will get media access easily. The easy access to media without awareness could lead to wrong usage [64]. Creating awareness about STIs prevention, adding the educational program about STIs and its consequence in the media program could minimize the spread of STIs.

(Rule 8) indicated that if a person lives in Ethiopia except in Tigray region**,** has a wealth status of richest**,** is female, and who has media access, then there will be a 61.0% chance of being affected by STIs. This result indicated that if we avoid the early initiation of sexual practice related to work (Refer Rule 6), we can minimize the probability of STIs among those regions by 7% (Rule 6). This rule (Rule 6 and Rule 8) emphasizes how the predictors integrate one another in a hidden manner. It also gives clear, detailed, specific and evidential information for policymakers.

(Rule 9) indicated that if a person chews chat and lives in Benishangul, SNNPR, Gambela, Harari or Addis Ababa, then there will be a 57% chance of being affected by STIs. This result pointed out that in these regions, chat chewing was the main factor that affects the prevalence of STIs. So, preventing the availability of chat in these regions could minimize the spread of STIs.

### Strength and limitation of the study

This study used a national representative EDHS 2016 dataset. It covers almost all risky group of the population which is vulnerable to STIs. In this research, the researchers applied the two most important statistical analysis models (spatial analysis and machine learning prediction) and dig out the hidden information about STI predictors and its association. This study applied MLA to GIS to generate evidences on spatial distributions and predictors of STIs to inform policymakers for STIs prevention and control. This analysis identified the key factors associated with STIs in a specific area and magnitude. However, this study was not free from limitations as the study used secondary data, the analysis did not include important STI risk factors. The prevalence of STIs in this study was based on the self-report of STI and its symptoms which were not validated by any medical practitioner through testing. Hence, information provided might be subject and vulnerable to information bias due to asymptomatic nature of most STIs and also respondents with STIs may feel afraid or ashamed to declare having STIs.

## Conclusion

The spatial distribution of STI among sexually active men and women in Ethiopia was not random rather it was clustered, with the highest rates being located in the border of Southwest Oromia and Northern part of SNNR, Border of Southwest Amhara and Northwest Oromia region and entire Harari Regional State.

Applying random forest machine learning algorithm for STI prediction in Ethiopia is the proposed model to identify the predictors of STIs. The random forest algorithm model pointed out the factors such as region, wealth, age, educational level, age at first sex, working status, marital status, media access, alcohol, chat, and sex of respondent with different combination. This leads to a proffer policy direction regarding the prevention and control of STI in Ethiopia.

Depending on their contribution and its association, those identified factors (predictors) were different in different area. The intervention, preventing and controlling strategies for STIs in Ethiopia should not be the same. This association results showed as case-effect relationship between the predictors or factors and the disease in detail and specific manner. This is used to dig out the hidden information about the cause and disease relation that could not addressed by classical statistical analysis method like logistic regression. Therefore, the concerned governmental and non-governmental organization should be applying different intervention mechanism in different risky area according to this research results.

### Recommendation

Based on the finding of this study the following recommendation were forward by the researcher for the following recommendation. Future researchers focused on clinical result dataset rather than self- reported data for machine learning prediction of STI. Future works needs to implement disease prediction application based on the clinically evident symptoms.

Policy makers should be considering this research result and propose STI intervention or controlling mechanism in Ethiopia and the policies should different in different area of the country depend on the factors (predictors) relationship with STI.

## Data Availability

The data that support the findings of this study are available from [DHS website (https://dhsprogram.com/data/) but restrictions apply to the availability of these data, which were used under license for the current study, and so are not publicly available. Data are however available from the authors upon reasonable request and with permission of [DHS (ICF)] program.

## Abbreviations

AIDS: Acquired immunodeficiency syndrome
AOR: Adjusted odds ratio
ART: Antiretroviral treatment
AUC: Area under curve
BV: Bacterial vaginosis
CDC: Centers for Disease Control
CI: Confidence interval/credible interval
DHS: Demographic and Health Survey
EA: Enumeration area
EDHS: Ethiopia Demographic and Health Survey
ESRI: Environmental Systems Research Institute
GIS: Geographic Information System
GPS: Global positioning system
GUD: Genital ulcer disease
HBV: Hepatitis B is a vaccine
HIV: Human immunodeficiency virus
HSV2: Herpes Simplex Virus Type 2
ICF: International Classification of Functioning
PHC: Primary Housing Census
ROSE: Random over sampling example
SMOTE: Systematic minority over sampling technique
SSA: Sub-Sahara Africa
STI: Sexually transmitted infection
WHO: World Health Organization
